# Efficacy of Biologic Therapies in the Management of Allergic Rhinitis: A Systematic Review

**DOI:** 10.7759/cureus.71408

**Published:** 2024-10-14

**Authors:** Ibtihal Yamani, Khulud Bu Saeed, Amjaad Alsulami, Salam Sait, Abdulaziz H Althumali

**Affiliations:** 1 Otolaryngology - Head and Neck Surgery, King Abdullah Medical City, Makkah, SAU; 2 Otolaryngology - Head and Neck Surgery, Alhada Armed Forces Hospital, Taif, SAU

**Keywords:** allergic rhinitis, biologic therapies, dupilumab, omalizumab, outcomes

## Abstract

Allergic rhinitis (AR) is a common chronic condition characterized by nasal congestion, sneezing, and itching, which significantly impacts quality of life. Traditional treatments, including antihistamines and intranasal corticosteroids, often fall short in managing moderate-to-severe cases. Recently, biologic therapies such as omalizumab and dupilumab have emerged as potential alternatives. This systematic review aims to evaluate the efficacy and safety of these biologic therapies in the management of AR.

A comprehensive literature search was conducted across PubMed, Embase, Cochrane Library, and Web of Science to identify studies published between 2000 and 2024. Studies included were randomized controlled trials, cohort studies, and post-hoc analyses that assessed the impact of biologics on AR symptoms. Data on study characteristics, population demographics, intervention details, and outcomes were extracted and analyzed.

The review included nine studies evaluating omalizumab and dupilumab. Omalizumab demonstrated significant improvements in nasal symptoms and quality of life, with notable efficacy in reducing symptoms and improving asthma control in patients with moderate-to-severe AR. Dupilumab also showed positive outcomes, particularly in patients with comorbid asthma and perennial AR, by reducing severe exacerbations and improving symptom scores.

Biologic therapies, including omalizumab and dupilumab, offer promising alternatives for the management of AR, especially in cases that are severe or refractory to conventional treatments. The evidence supports their efficacy in improving symptoms and quality of life. Nevertheless, further research is required to address the limitations identified, including the need for long-term data and clarification of the mechanisms of action. These findings underscore the potential of biologics in advancing the treatment of AR and highlight the importance of ongoing research to optimize patient outcomes.

## Introduction and background

Allergic rhinitis (AR) is a prevalent chronic condition characterized by symptoms such as nasal congestion, sneezing, itching, and rhinorrhea, significantly affecting the quality of life for millions of people worldwide [1-3]. The pathophysiology of AR involves a complex interaction between environmental allergens and the immune system, leading to the release of inflammatory mediators, including histamine and cytokines, which drive the allergic response [4,5]. Traditional management strategies for AR have centered around pharmacological treatments like antihistamines, intranasal corticosteroids, and allergen immunotherapy [6,7]. While these treatments can provide symptomatic relief, they are often limited in efficacy, particularly in patients with moderate-to-severe disease, and may not fully address the underlying immune dysregulation [7].

In recent years, biologic therapies have emerged as a promising alternative in the management of allergic diseases, including AR [8,9]. Biologics are targeted treatments that specifically interfere with key immunological pathways, offering the potential to reduce inflammation and modulate immune responses more effectively than conventional therapies [10,11]. Several biologic agents, such as monoclonal antibodies targeting IgE (omalizumab), IL-5 (mepolizumab), and IL-4/IL-13 (dupilumab), have shown efficacy in other allergic conditions like asthma and atopic dermatitis [12]. Given the shared immunological mechanisms between these diseases and AR, biologic therapies are increasingly being investigated for their potential role in managing AR, especially in patients with severe or refractory forms of the condition.

This systematic review aims to evaluate the current evidence on the efficacy of biologic therapies in the management of AR. By synthesizing data from clinical trials and observational studies, we aim to provide a comprehensive assessment of how biologics compare to conventional treatments in terms of symptom control, quality of life improvement, and safety profile. The findings of this review will help inform clinical decision-making and identify gaps in the literature where further research is needed to optimize the use of biologics in AR.

## Review

Methodology

Search Strategy

This systematic review was conducted following the Preferred Reporting Items for Systematic Reviews and Meta-Analyses (PRISMA) guidelines. A comprehensive literature search was performed across multiple electronic databases, including PubMed, Embase, Cochrane Library, and Web of Science, to identify relevant studies published between 2000 and 2024. The search strategy was designed to capture clinical trials, observational studies, and randomized controlled trials that evaluated the efficacy of biologic therapies in the management of AR. Keywords and medical subject headings terms related to "allergic rhinitis," "biologic therapies," and specific biologics, such as "omalizumab," "mepolizumab," and "dupilumab," were used to ensure a thorough search.

Eligibility Criteria

The inclusion criteria were defined to select studies that (1) involved human subjects diagnosed with AR, (2) evaluated the efficacy of biologic therapies compared to placebo or conventional treatments, (3) reported clinical outcomes such as symptom relief, quality of life, and safety profiles, and (4) were published in English. Studies focusing exclusively on other allergic conditions, without specific analysis of AR, were excluded. Additionally, case reports, reviews, editorials, and conference abstracts were not considered in this review.

Data Extraction

Data extraction was performed independently by two reviewers, who screened titles and abstracts for relevance, followed by a full-text review of potentially eligible studies. Discrepancies between reviewers were resolved through discussion and, if necessary, consultation with a third reviewer. Extracted data included study characteristics, sample size, patient demographics, biologic agent used, duration of treatment, primary and secondary outcomes, and adverse events.

Quality Assessment and Data Synthesis

The quality of the included studies was assessed using the Cochrane Risk of Bias Tool for randomized controlled trials and the Newcastle-Ottawa Scale for observational studies. Studies were rated as high, moderate, or low quality based on criteria such as randomization, allocation concealment, blinding, and completeness of outcome data. A narrative synthesis of the results was performed, and where appropriate, a meta-analysis was conducted to quantify the pooled effect of biologic therapies on AR symptoms and quality of life.

Studied Outcomes

The primary outcomes of interest were symptom reduction, as measured by validated tools such as the total nasal symptom score (TNSS) and rhinoconjunctivitis quality of life questionnaire (RQLQ). Secondary outcomes included the frequency of exacerbations, use of rescue medications, and adverse events associated with biologic therapies. Results were presented in a descriptive manner, with summary tables providing an overview of study characteristics and outcomes.

Results

**Figure 1:**
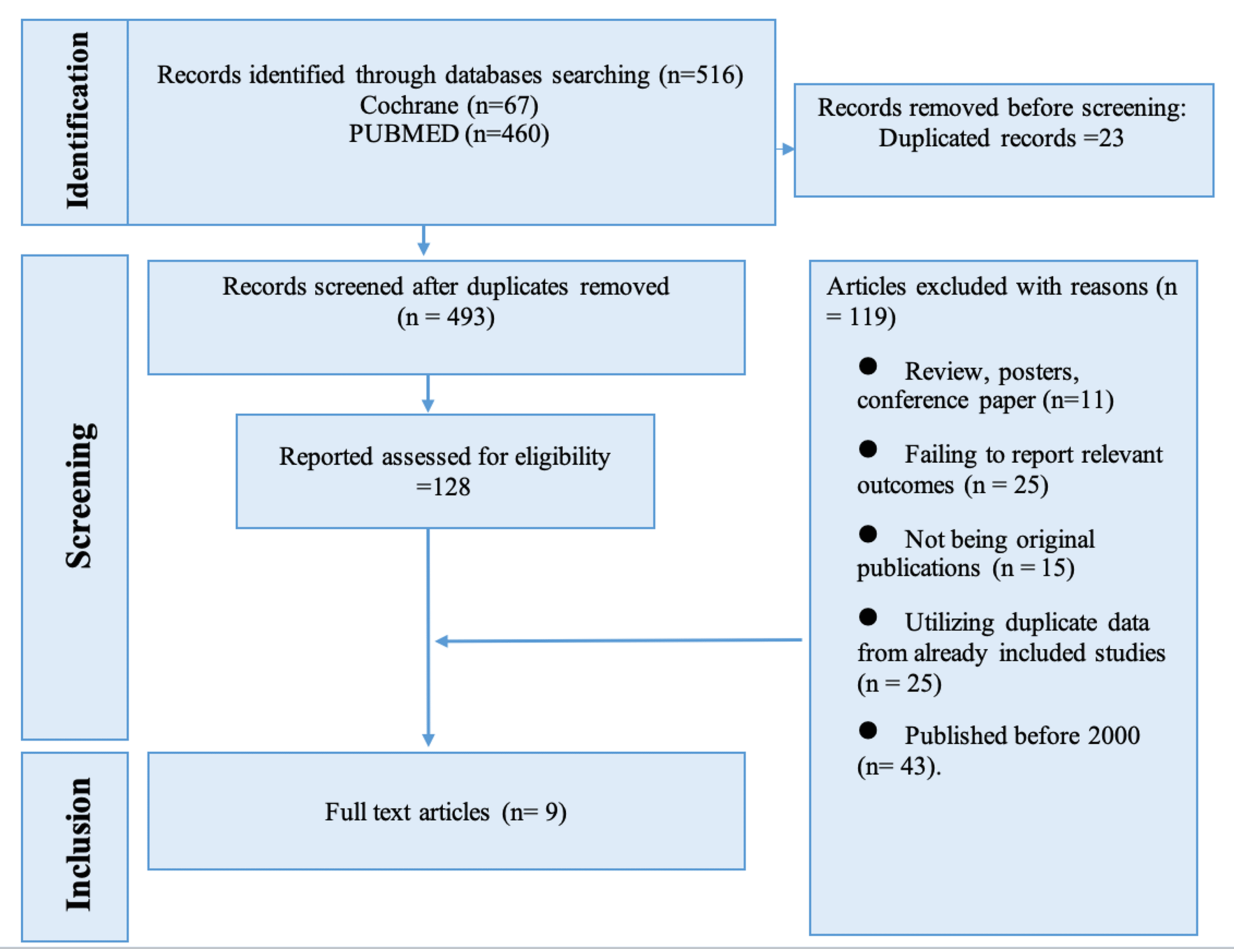
PRISMA figures showing the steps to choose the studies for systematic review PRISMA: Preferred Reporting Items for Systematic Reviews and Meta-Analyses

This review included nine studies that are consistent with the inclusion and exclusion criteria (Figure 1). The results of this systematic review are summarized into three main categories: general characteristics of the included studies, population characteristics, intervention and control groups, and the outcomes observed.

Study Characteristics

The review incorporated various study designs, including randomized controlled trials, prospective and retrospective cohort studies, and post hoc analyses [13-21]. The studies were conducted across different countries, including Italy, China, the USA, Germany, and others, with sample sizes ranging from 11 to 1,902 participants and follow-up durations spanning from a few weeks to several years. The primary objective of these studies was to evaluate the efficacy and safety of biologic therapies, such as omalizumab and dupilumab, in managing AR and associated conditions (Table 1).

**Table 1:** General characteristics of the studies SIT: specific immunotherapy, FEV1: forced expiratory volume in 1 second, SNOT-22: sino-nasal outcome test-22, AR: allergic rhinitis, SCIT: subcutaneous immunotherapy

Author (s)	Year	Study design	Country	Sample size	Follow-up duration	Objective
Cavaliere et al. [13]	2020	Prospective Cohort	Italy	11	36 months	To evaluate the effect of omalizumab on AR and asthma
Ma et al. [14]	2021	Retrospective Cohort	China	60	2.9 months	To evaluate the effect of omalizumab on seasonal AR
Casale et al. [15]	2001	Randomized Controlled Trial	USA	536	12 weeks	To evaluate the effect of omalizumab in moderate-to-severe AR during the pollen season
Kamin et al. [16]	2010	Randomized, double-blind, placebo-controlled, multi-center trial	Germany	221	24 weeks	To evaluate the effect and tolerability of SIT combined with omalizumab versus placebo in children with seasonal AR
Li et al. [17]	2020	Post-hoc analysis of Phase III randomized controlled study	China	608	Not specified	To evaluate the effect of omalizumab in moderate-to-severe allergic asthma patients
Busse et al. [18]	2020	Randomized controlled trial	Not specified	1902	52 weeks	To evaluate the effect of dupilumab on asthma exacerbations and FEV1 in patients with comorbid perennial AR
Weinstein et al. [19]	2018	Post hoc analysis of Phase 2b study	Not specified	776	Not specified	To evaluate the effect of dupilumab on SNOT-22 scores and AR-associated symptoms in asthma patients with comorbid perennial AR
Kamal et al. [20]	2021	Randomized controlled trial	Not specified	103	16 weeks	Investigate the pharmacokinetics and concentration-response relationships of dupilumab in combination with SCIT for grass pollen-induced seasonal AR
Nettis et al. [21]	2022	Multicenter, observational, prospective study	Italy	82	Not specified	To evaluate the effect and safety of dupilumab in adult patients with chronic rhinosinusitis with nasal polyps and comorbid conditions

Population and Intervention Details

The populations studied varied, with some studies focusing on patients with seasonal AR, while others included those with chronic rhinosinusitis or comorbid asthma. Interventions primarily involved biologic therapies, such as omalizumab and dupilumab, administered either alone or in combination with other treatments like subcutaneous immunotherapy (SCIT). Control groups typically received a placebo or standard care, allowing for a comparative analysis of the biologic agents' effectiveness (Table 2).

**Table 2:** Included population, intervention, and control groups SIT: specific immunotherapy, SCIT: subcutaneous immunotherapy, AR: allergic rhinitis

Study	Population characteristics	Intervention group	Control group
Cavaliere et al. [13]	11 patients (7 males, 4 females) with persistent AR and poorly controlled asthma	Omalizumab (every 4 weeks, subcutaneous injections)	No control group
Ma et al. [14]	60 patients (25 males, 35 females) with seasonal AR (21 with co-existing asthma)	Omalizumab (varied dosages, median 150 mg)	No control group
Casale et al. [15]	536 patients aged 12-75 with moderate to severe seasonal AR	Omalizumab (50 mg, 150 mg, or 300 mg, subcutaneous injections)	Placebo group (administered saline solution)
Kamin et al. [16]	Children with seasonal AR	SIT (grass or birch pollen) + omalizumab or placebo	SIT (grass or birch pollen) + placebo
Li et al. [17]	Chinese patients with moderate-to-severe allergic asthma	Omalizumab	Placebo
Busse et al. [18]	Patients with uncontrolled, moderate-to-severe asthma with comorbid perennial AR	Dupilumab (200 mg or 300 mg every 2 weeks)	Placebo
Weinstein et al. [19]	Patients with asthma and comorbid perennial AR	Dupilumab (200 mg or 300 mg every 2 weeks)	Placebo
Kamal et al. [20]	Patients with grass pollen-induced seasonal AR	Dupilumab (300 mg every 2 weeks) + SCIT	SCIT only
Nettis et al. [21]	Adult patients with chronic rhinosinusitis with nasal polyps	Dupilumab (doses not specified)	Not applicable (observational)

Outcomes and Key Findings

The review highlighted significant findings related to the efficacy of biologic therapies in improving AR symptoms and overall quality of life. For instance, studies involving omalizumab demonstrated notable improvements in nasal symptoms and quality of life. Cavaliere et al. reported significant enhancements in both rhinitis and asthma symptoms [13], while Casale et al. found that omalizumab significantly reduced nasal symptom severity and improved quality of life compared to placebo [15]. Ma et al. observed improvements in the RQLQ and TNSS, with an 83.3% response rate [14]. The studies involving dupilumab also yielded positive results. Busse et al. found that dupilumab reduced severe asthma exacerbations and improved FEV1, particularly in patients with elevated type 2 inflammatory biomarkers [18]. Weinstein et al. reported that dupilumab improved SNOT-22 scores and AR-associated symptoms, although the 200 mg dose showed a numerical but not statistically significant improvement compared to the 300 mg dose [19]. Kamal et al. noted that dupilumab in combination with SCIT did not significantly alter serum dupilumab concentrations but did increase specific IgG4 levels [20]. In terms of safety, the studies generally reported good tolerability of biologic therapies. Kamin et al. noted a slight increase in adverse events associated with omalizumab, though no severe reactions such as anaphylaxis were observed [16]. Nettis et al. found significant improvements in various efficacy measures for chronic rhinosinusitis with nasal polyps and comorbid conditions, with the treatment being well-tolerated overall [21]. These findings collectively suggest that biologic therapies offer a promising alternative for managing AR, particularly in patients with severe or refractory symptoms. However, variations in study design, patient populations, and outcome measures highlight the need for further research to optimize treatment strategies and establish long-term efficacy and safety profiles (Table 3).

**Table 3:** Outcomes and results SIT: specific immunotherapy, FEV1: forced expiratory volume in 1 second, AQLQ: asthma quality of life questionnaire, ACQ: asthma control questionnaire, GETE: global evaluation of treatment effectiveness, SNOT-22: 22-item sino-nasal outcome test, AR: allergic rhinitis, SCIT: subcutaneous immunotherapy, NPS: nasal polyps, CRSwNP: chronic rhinosinusitis without nasal polyps, RCSS: rhinitis control scoring system, RQLQ: rhinoconjunctivitis quality of life questionnaire, EASI: eczema area and severity index, DLQI: dermatology life quality index, AD: atopic dermatitis, UAS7: urticaria activity score, VAS: visual analog scale, ACT: asthma control test, TNSS: total nasal symptom score, QOL: quality of life

Study	Primary outcome	Secondary outcomes	Key findings
Cavaliereet al. [13]	Improvement in VAS for rhinitis and ACT	Lung function, nasal endoscopy, biomarkers (eosinophils, neutrophils, FeNO)	Significant improvement in both rhinitis and asthma symptoms; no side effects recorded
Ma et al. [14]	Improvement in RQLQ and TNSS	ACT	Improved quality of life, nasal symptoms, and asthma control; 83.3% response rate
Casale et al. [15]	Reduction in nasal symptom severity	Reduced antihistamine use and improved rhinitis-specific QOL	300 mg omalizumab significantly reduced nasal symptoms and improved QOL compared to placebo
Kamin et al. [16]	Tolerability of SIT + omalizumab vs. placebo	Local reactions, gastrointestinal and ear symptoms	Good tolerability overall; a slight increase in adverse events in omalizumab groups; no anaphylaxis
Li et al. [17]	Improvements in FEV1, AQLQ, ACQ, and GETE scores	Baseline IgE levels, eosinophil count, allergen profile	Greater efficacy of omalizumab in patients with high baseline IgE and multiple allergens
Busse et al. [18]	Severe asthma exacerbation rates, FEV1 improvements	Asthma control, quality of life measures, type 2 inflammatory biomarkers	Dupilumab reduced severe exacerbations and improved FEV1; greater efficacy in patients with elevated type 2 biomarkers
Weinstein et al. [19]	SNOT-22 total score, AR-associated symptoms	Not specified	Dupilumab 300 mg q2w improved SNOT-22 scores and AR-associated symptoms; 200 mg showed numerical but not significant improvement
Kamal et al. [20]	Functional serum dupilumab concentrations, TG-specific IgE and IgG4	TG-specific IgE and IgG4 concentrations	SCIT + dupilumab increased TG-specific IgG4 but did not alter dupilumab concentrations; similar results in both groups
Nettis et al. [21]	SNOT-22 score, NPS score for CRSwNP, RCSS, RQLQ, FEV1, AQLQ	EASI, DLQI for AD, UAS7 for chronic urticaria	Significant improvements in SNOT-22, NPS, and other efficacy measures for CRSwNP and comorbidities; well-tolerated

Discussion

The findings of this systematic review provide compelling evidence supporting the efficacy of biologic therapies in the management of AR, particularly in patients with moderate to severe forms of the disease or those with comorbid conditions like asthma and chronic rhinosinusitis. The studies included in this review highlight improvements in both primary and secondary outcomes, such as symptom reduction, quality of life enhancement, and reductions in asthma exacerbations, underscoring the potential of biologic therapies to address the limitations of conventional AR treatments.

Omalizumab

Biologic agents like omalizumab and dupilumab demonstrated significant efficacy in reducing allergy responses across a range of studies [8,12,17,22-24]. Omalizumab, an anti-IgE monoclonal antibody [25,26], was particularly effective in reducing nasal symptoms and improving overall quality of life in AR patients, as seen in studies by Casale et al. and Ma et al. (2021) [14,15]. Omalizumab’s ability to inhibit IgE-mediated allergic responses has been well-established, and its use in AR aligns with previous studies showing its benefits in asthma and chronic idiopathic urticaria [27,28]. The study by Casale et al. further highlighted the efficacy of omalizumab in reducing nasal symptoms during the pollen season, with a significant reduction in the use of antihistamines and a marked improvement in rhinitis-specific quality of life scores [15]. Similarly, Cavaliere et al. observed substantial improvements in both rhinitis and asthma symptoms, further indicating omalizumab's dual benefit in managing comorbid conditions [13].

Dupilumab

Dupilumab, which targets the IL-4 and IL-13 pathways involved in type 2 inflammation [29-31], also showed promising results in this review. Busse et al. demonstrated that dupilumab significantly reduced asthma exacerbations and improved FEV1 in patients with concomitant AR, particularly in those with elevated type 2 biomarkers [18]. These findings are consistent with the growing body of literature supporting the role of dupilumab in managing type 2 inflammatory diseases, including atopic dermatitis and asthma [32-34]. Weinstein et al. further demonstrated the utility of dupilumab in improving SNOT-22 scores and AR-associated symptoms in asthma patients with perennial AR [19]. This suggests that dupilumab could be a valuable therapeutic option for AR patients who do not achieve adequate control with conventional therapies, particularly those with comorbidities that exacerbate their allergic symptoms.

Biologics + SCIT

The combination of biologic therapies with other treatment modalities, such as SCIT, was also explored. Kamin et al. evaluated the tolerability of omalizumab combined with SCIT and reported generally good tolerability, though there was a slight increase in adverse events compared to placebo [16]. While this combination therapy has shown potential in enhancing immune tolerance to allergens, further research is necessary to better understand the long-term safety and efficacy of combining biologics with SCIT in treating AR [35].

In terms of safety, biologic therapies were generally well tolerated across the studies included in this review. While some studies, such as that by Kamin et al., reported a slight increase in adverse events with biologic therapy, no severe reactions, such as anaphylaxis, were observed [16]. This is an important consideration, as the safety profile of biologics is a critical factor in their long-term use, particularly in vulnerable populations such as children and those with multiple allergic conditions [36].

Limitations of the study

Despite the promising results, there are several limitations worth mentioning. Many of the studies included had variations in follow-up duration, sample size, and study design, which could introduce heterogeneity in the outcomes observed. Additionally, the majority of the studies focused on short- to medium-term outcomes, with few addressing the long-term efficacy and safety of biologic therapies in AR. Future research should aim to address these gaps by conducting larger, longer-term studies that evaluate the sustained efficacy and safety of biologic agents, particularly in patients with severe or refractory AR.

Moreover, the cost of biologic therapies remains a significant barrier to widespread use. As these treatments are often more expensive than conventional pharmacotherapy, cost-effectiveness analyses are needed to assess the financial feasibility of incorporating biologics into routine clinical practice for AR [37]. While the clinical benefits of biologics are evident, their cost may limit access, especially in low-resource settings. Therefore, healthcare systems must evaluate the potential impact of biologics on overall healthcare costs, balancing efficacy with economic considerations.

## Conclusions

This systematic review demonstrates that biologic therapies, particularly omalizumab and dupilumab, offer significant benefits in managing AR, particularly in patients with moderate to severe disease or comorbid conditions like asthma. These agents show promise in reducing symptoms, improving quality of life, and enhancing asthma control. While biologics are generally well-tolerated, further research is required to evaluate their long-term safety and cost-effectiveness. As biologic therapies become increasingly integrated into AR management, clinicians must consider patient-specific factors such as disease severity, comorbidities, and economic feasibility when deciding on the most appropriate treatment strategy.
